# Environmental disturbance increases social connectivity in a passerine bird

**DOI:** 10.1371/journal.pone.0183144

**Published:** 2017-08-30

**Authors:** Samantha M. Lantz, Jordan Karubian

**Affiliations:** Department of Ecology and Evolutionary Biology, Tulane University, New Orleans, Louisiana, United States of America; University of Oxford, UNITED KINGDOM

## Abstract

Individual level response to natural and anthropogenic disturbance represents an increasingly important, but as yet little understood, component of animal behavior. Disturbance events often alter habitat, which in turn can modify behaviors of individuals in affected areas, including changes in habitat use and associated changes in social structure. To better understand these relationships, we investigated aspects of habitat selection and social connectivity of a small passerine bird, the red-backed fairywren (*Malurus melanocephalus*), before vs. after naturally occurring fire disturbance in Northern Territory, Australia. We utilized a social network framework to evaluate changes in social dynamics pre- vs. post-fire. Our study covered the non-breeding season in two consecutive years in which fires occurred, and individuals whose habitat was affected and those that were not affected by fire. Individuals in habitat affected by fires had stronger social ties (i.e. higher weighted degree) after fires, while those that were in areas that were not affected by fire actually had lower weighted degree. We suggest that this change in social connections may be linked to habitat. Before fires, fairywrens used habitat that had similar grass cover to available habitat plots randomly generated within our study site. Fire caused a reduction in grass cover, and fairywrens responded by selecting habitat with higher grass cover relative to random plots. This study demonstrates how changes in habitat and/or resource availability caused by disturbance can lead to substantive changes in the social environment that individuals experience.

## Introduction

Social structure shapes processes ranging from disease and parasite transmission [1,2], to information transfer [3,4], to differential access to resources such as food and mates [5]. For this reason, non-random associations between individuals are key drivers of many ecological and evolutionary processes [6,7]. The underlying factors that promote non-random associations, and changes among these associations, are not well understood. Although theoretical studies have provided important insights into the general relationships between environmental variables and social organization (e.g., [8]), our knowledge of the specific factors that shape variation in sociality across and within systems remains incomplete [9]. One potential influence on variation in social structure is the ecological environment, for example habitat structure or complexity, which has been examined extensively in laboratory settings (e.g. [10]), and less commonly in natural environments [11,12]. As such, a more thorough understanding of how environmental variation impacts social structure is a clear goal in behavioral ecology [9,13,14].

Social organization can vary in different ecological environments, as measured along a gradient or in discrete habitats (e.g., [15,16]). Relatively open habitats are often associated with a higher frequency of interactions because of increased predation risk [17] or because structural complexity itself may restrict social contact [18]. Alternatively, higher structural complexity may increase social connectivity by reducing potential pathways or restricting individuals to certain areas [12] such that individuals in those areas interact more. A third alternative is that social structure may be consistent across different habitats[16]. These and other studies characterizing the relationships between habitat structure and sociality represent important advances in our understanding of social behavior, but do not account for the fact that animals often inhabit dynamic environments, in which habitat parameters change over time, sometimes abruptly. Changes in available habitat or the distribution of resources may result in individuals shifting resource use, which can either increase [11]or decrease [19] social interactions. Dynamic environments may also be associated with fission-fusion sociality, with different patterns of sociality depending on changing habitat [20,21] or predation risk [22]. Changes in social interactions as a result of a dynamic environment are important because of the potential to influence fitness. Increases in social interactions can increase competition, aggressive interactions, and/or parasite transmission [11] or can change the potential for sexual selection [23].

Most studies focus on comparisons between populations in different habitats or in control vs. experimental groups after a modification of habitat complexity or environmental resources, rather than direct before and after comparisons of the same population. For example, studies have investigated social organization in lizard populations that have experienced different fire regimes [24], or compared lizard populations with and without experimentally-added habitat complexity [12]. However, both of these studies compare populations in different environments to assess the influence on social structure, but do not investigate the change within these populations across multiple time points. Studying the same population before and after a change or disturbance event offers a more direct measure of how habitat disturbance influences social structure, and thus is expected to increase our understanding of the drivers of sociality. Much as social structure can vary across different types of habitat or resource availability, we might similarly expect changes in habitat or resource availability to impact social structure among resident species that remain in an area before, during and after environmental change. In one such example, bottlenose dolphins (*Tursiops aduncus*) living in the same area altered their social organization in response to changes in food availability caused by the presence vs. absence of prawn trawlers [25,26]. However, few studies have directly addressed how social structure varies before vs. after naturally occurring habitat disturbances, as mediated by alteration in habitat structure and resource availability. It is reasonable to expect that individual behavior and resultant social organization would be similar in populations facing environmental disturbances to those in discrete habitats following disturbance or variation. However, there is little information to support this idea, in part because of the difficulty of using targeted experimental approaches to compare population social structure before vs. after disturbances.

In this study, we examined the social associations of red-backed fairywrens (*Malurus melanocephalus*) in northern Australia, in the context of bush fires that produced rapid changes to their environment. Red-backed fairywrens are small insectivorous passerine birds that are residents of fire-prone tropical savannas [27]. Red-backed fairywrens are a fitting species in which to study how habitat disturbance mediates social density because they depend on grass to forage and for protection from predators, and are often absent from recently burned areas for weeks or months [28–31]. Also, red-backed fairywrens form loose flocks of varying size and demographic composition during the non-breeding season, when the current study was conducted [27]. We used a social network framework to quantify how bush fires influence red-backed fairywren social associations, and investigated changes in habitat as a potential mechanism that could influence sociality. We predictedthat individuals directly impacted by fire would have increased social connectivity following fires because they would congregate in higher densities in areas with remaining vegetation. Alternatively, we might expect fragmentation of grass habitat caused by fire to limit social interactions, for example by limiting movement between isolated patches of suitable habitat. By comparing individual habitat preferences and social connections both before and after fires, we were able to directly assess the impact of habitat disturbance in social density in this system.

## Methods

### Ethics statement

This study was carried out in accordance with the recommendations of the Animal Ethics Committee at Charles Darwin University and the Institutional Animal Care and Use Committee at Tulane University. The protocol was approved by both the Animal Ethics Committee at Charles Darwin University, Darwin, Australia (Permit Number: A12032), and by the Institutional Animal Care and Use Committee at Tulane University (Permit Number: 0395). This study was carried out on private property with permission from the land owner.

### Study system and fire history

**Fig 1:**
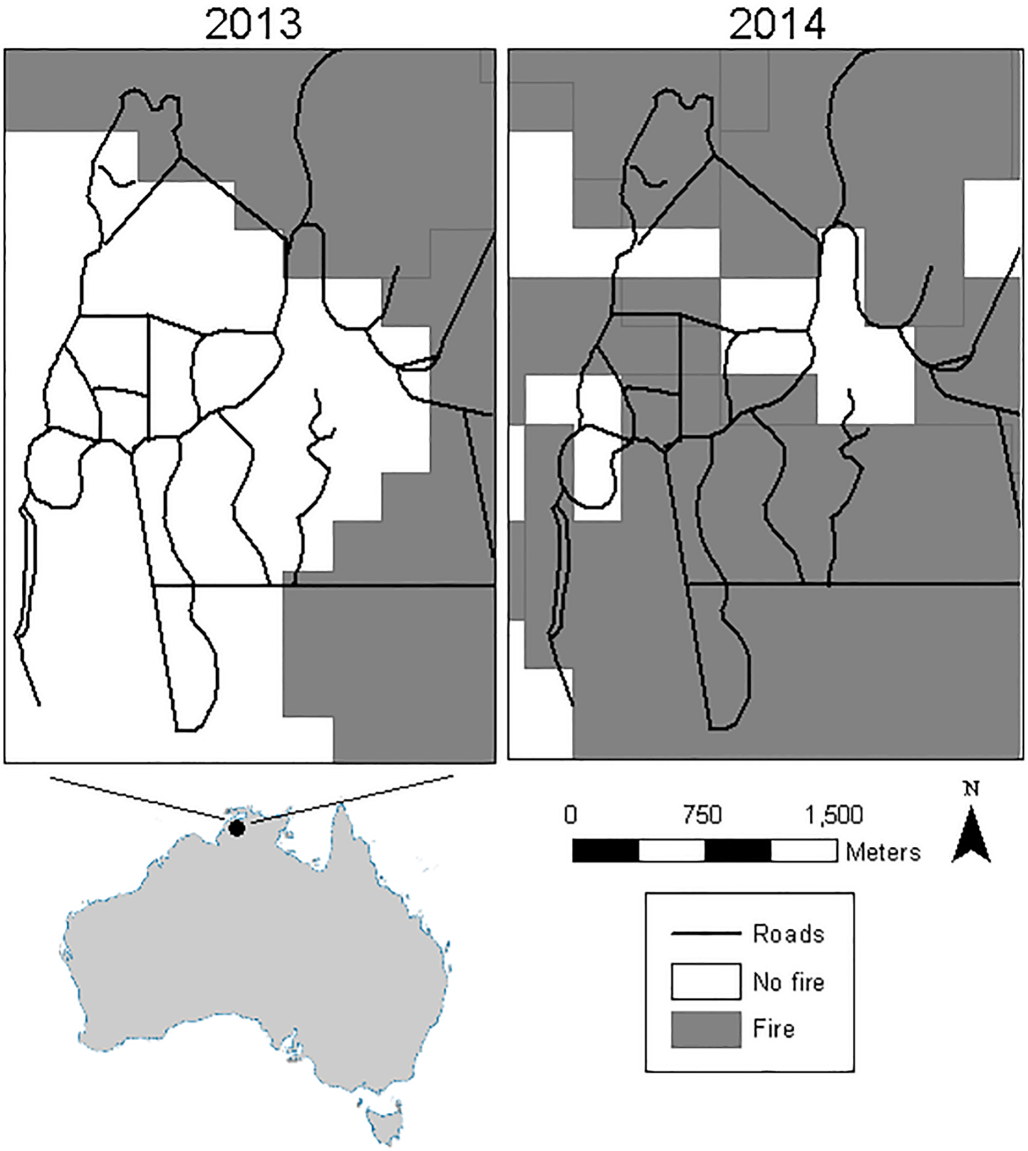
Fire scars from 2013 and 2014. Fire scars from the Northern Australia Fire Information network (NAFI, http://www.firenorth.org.au/nafi3/). In both years, fires in July burned a large portion of the field site. Map of Australia from https://www.cia.gov/library/publications/the-world-factbook/geos/as.html.

We studied a color-banded population of red-backed fairywrens on Coomalie Farm (13°02’ S, 131°02’ E), located approximately 87 km south of Darwin in Northern Territory, Australia, from May-Aug 2013 and 2014. During each field season, we captured or re-captured adult red-backed fairywrens marked them with individually specific colored leg bands and an Australian Bird and Bat and Banding Scheme (ABBBS) numbered aluminum band, as part of a larger ongoing study of sociality and signal acquisition in this species(see [32]). Population monitoring began in 2012 with consistent banding across years, meaning that the majority of birds in the population were colorbanded. Our study period coincided with the nonbreeding season for red-backed fairywrens and the dry season in the Northern Territory. The habitat at the study site consists of a mosaic of savanna and open woodland habitats, and climate is characterized by alternating wet and dry seasons, with periods of high rain and flooding from October to April, and little rain and many bush fires from May to September. Although the property owner at Coomalie frequently performs small controlled burns (several hectares) that serve as fire breaks, large uncontrolled fires occurred in late July in both years of the study. In 2013, a large fire burned 30% of the approximately 700 ha study site and in 2014 multiple fires burned approximately 80% of the study site (Fig 1). In 2014, a small fire burned the northern portion of the field site in late June, and a larger fire burned the majority of the site in mid-July. Fires in both years also burned thousands of surrounding ha. In both years of the study, fires occurred midway through the study period, which allowed us to collect data on fairywren social behavior both before and after the fires. We compared pre-and post-burn at an unburned portion of the site to serve as a control in one of the years, and also collected data on habitat before and after fires in one year. To characterize the spatial extent of the fires each year, we obtained georeferenced fire scars from the Northern Australia Fire Information network (NAFI, http://www.firenorth.org.au/nafi3/). NAFI maps fire scars in real time, and summarizes fire scars each month at a 250-m resolution.

### Social observations and fairywren response to fire

We measured social associations of color-banded birds through opportunistic group observations of colorband resightings and capture of banded individuals in mist nets. We defined a social interaction as an observation of a group of individuals that were moving and vocalizing together in a cohesive manner, generally within a radius of ~10 m. Our observations used the ‘gambit of the group’ assumption [33] that that all individuals in a group were associating with all other members of the group. We walked all roads and trails within the study site at least once per week, as well as covering areas between roads. Because of the large number of roads and trails throughout the study site, and because this population has been color-banded and studied since 2012, we are confident that our opportunistic sightings provided an accurate picture of social interactions on the site during the study period. We also considered individuals to be in the same group if they were captured in the same mist net at the same time. We conducted active mist netting specifically focused on this species, so it was always clear that individuals captured together were in the same group. We used a Garmin GPS to mark the point where opportunistic sightings or mist net captures occurred, and compared these spatial locations to fire scars.

We mapped GPS locations of bird sightings and captures along fire scars to assess which birds were directly impacted by fire in each year, using resightings from both 2013 and 2014. In 2013, we considered individuals that we consistently sighted in locations that were directly burned or within 100 m of the fire scar to be potentially affected by the fire, and those that were sighted at least 500 m from the fire scar as unaffected. Fairywrens are relatively weak fliers [27] with small home ranges [34], thus 500 m is an suitable buffer between zones. Importantly, we did not have birds move between affected and unaffected areas, and we did not include any birds in this study that were located between 100–500 m from fire scars. Hereafter, we refer to fairywrens as either ‘affected’ or ‘unaffected’ by the fire in comparisons. We never observed interactions between affected vs. unaffected individuals, and because of the spatial separation between these groups we consider them independent for the purposes of analysis. In 2014, because of the large expanse of the fire on the study site (below), we considered all individuals to be affected by the fire (i.e., there were no birds that were consistently in areas more than 500 m from the fire scar or that would not have the opportunity to come into contact with individuals that would have been affected).

We used resighting and capture observations to create weighted social networks in R version 3.2.4 [35] using the packages igraph [36], sna [37], and asnipe [38]. Social networks use a system of nodes (color-banded individuals) connected by edges (relationships; in this case, edges mean that birds were seen or captured together). We included any individuals seen at least twice in our analyses. Our analyses focus on the effects of fire on weighted degree, which is a measure of association between individuals, ranging from 0 (never seen together) to 1 (always seen together). Weighted networks consider the proportion of times that individuals were seen together when calculating edge weights. Our behavioral observations did not attempt to distinguish who initiated interactions, thus our measure of degree is non-directional, and does not indicate the nature of the interaction (e.g. affiliative or agnostic, although most interactions involved individuals foraging together). Other social network metrics (e.g. individual centrality) were not informative because of the low density of fairywrens at this field site.

In order to separate potential temporal effects from those resulting from the fire, in 2013 we opportunistically used a before-after control-impact design (BACI [39]). We standardized our data collection periods so that we include sightings from an approximately 6-week period on either side of the fire, with 32 observation-days both before and after the fire (pre-fire: 15 May-28 June, post-fire: 13 July-24 August). We separately compare social networks before vs. after the fire with birds that were affected and not affected by the fire. In 2014, we compare weighted degree pre- vs. post-fire for birds affected in either the June or July fires, with dates varying based on the fire event (the smaller June fire or the larger July fire). Thus, for most birds the pre-fire sighting period (13 June-17 July) was slightly longer than the post-fire period (18 July-14 August). For birds affected by the June fire (n = 10), the pre-fire period was from 13 June-23 June and the post-fire period was 24 June-14 August; including or removing these 10 birds from our analyses did not qualitatively change our results, so we include them in order to boost our sample sizes. Our sightings included a larger number of individuals post-fire than pre-fire; restricting our analysis to only include the 32 individuals seen both before and after the fires did not change our qualitative results, thus we include all individuals seen in either period. We were not able to use the BACI design in 2014 because the large extent of the fire meant that there were no control areas after the fire.

Because network data violates assumptions of independence [40], we utilized network-appropriate permutations within the R package asnipe [38] to test for changes in sociality, specifically looking at how fire affected individual degree. In 2013, we created networks using group observations from four scenarios: 1) ‘pre-fire affected’, consisting of individuals spatially not affected by the fire in the time period before the fire, 2) ‘pre-fire not affected’, consisting of individuals spatially not affected by the fire in the time period before the fire, 3) ‘post-fire affected’, consisting of individuals spatially not affected by the fire in the time period after the fire, and, 4) ‘post-fire not affected’, consisting of individuals spatially not affected by the fire in the time period after the fire. We used group observations from each of these four scenarios to create group-by-interaction (GBI) matrices [41], and calculated the weighted degree of all individuals from association matrices created from the GBI data. We then used network permutations by swapping individuals between groups to create randomizations of the GBI data stream [41,42]. Individuals were only swapped between groups within the same treatment (i.e., affected individuals are only swapped with affected individuals). We made 1000 permutations of each of the four GBI matrices, and then recalculated the association matrices and the weighted degree for each permutation. We compared weighted degree to fire period (i.e., pre vs post) using linear models using data from both affected and not affected networks. To do so, we combined pre- and post-fire observations of all ‘affected’ birds into one dataframe and built a model comparing weighted degree pre- vs. post-fire. We repeated this process for groups ‘not affected’ by the fire. We then used data from network permutations to compare the coefficient for the magnitude of the slope in both models using the observed data to coefficients from null models created from each of the network permutations. We calculated P-values for one-tailed significance by comparing the observed slope relative to the distribution of slopes from the randomized data. In 2014, because of the large extent of the fire, we considered all birds to be affected, and thus were able to create a general linear model comparing weighted degree pre- vs. post-fire to a null model using the methods described above.

In both years, we also compared average group size for individuals pre- and post-fire, to determine the nature of any changes in weighted degree. For example, an increase in weighted degree could occur if an individual had social associations with more individuals or if that individual was more consistently sighted with the same group after a fire. To do so, we calculated average group size for each individual using the observed data and compared this group size pre- vs. post-fire, calculating significance by comparing the observed slope to the distribution of slopes from the permuted GBI data stream.

### Habitat response to fire

To assess changes in habitat in response to fire in 2014, we first characterized habitat that red-backed fairywrens used vs. all available habitat in our study area. We analyzed these data only for 2014 because we did not conduct post-fire vegetation surveys in 2013. We characterized vegetation at 'used' locations (i.e., locations in which birds were present on at least one occasion; N = 106) and 'random' locations (N = 152). We determined used locations via standardized transects and opportunistic sightings (above), and random locations by randomly generated points using all available coordinates within the transect area; random locations therefore represent habitat that was available to red-backed fairywrens, but that may or may not have been used. We focused our habitat measures on percent cover of grass because red-backed fairywrens rely on grass for cover from predators and as foraging substrate [27]. In each used and random plot, we measured the percent grass cover in 10 x 10 m plots to the nearest 5%. Because assumptions of normality were not met following standard transformations of the dependent variable grass cover, we used Wilcoxon rank sum tests to compare grass cover between used and available sites both before and after fire. This nonparametric approach did not allow us to test for an interaction between temporal period and use.

## Results

### Sociality change due to fire

**Fig 2:**
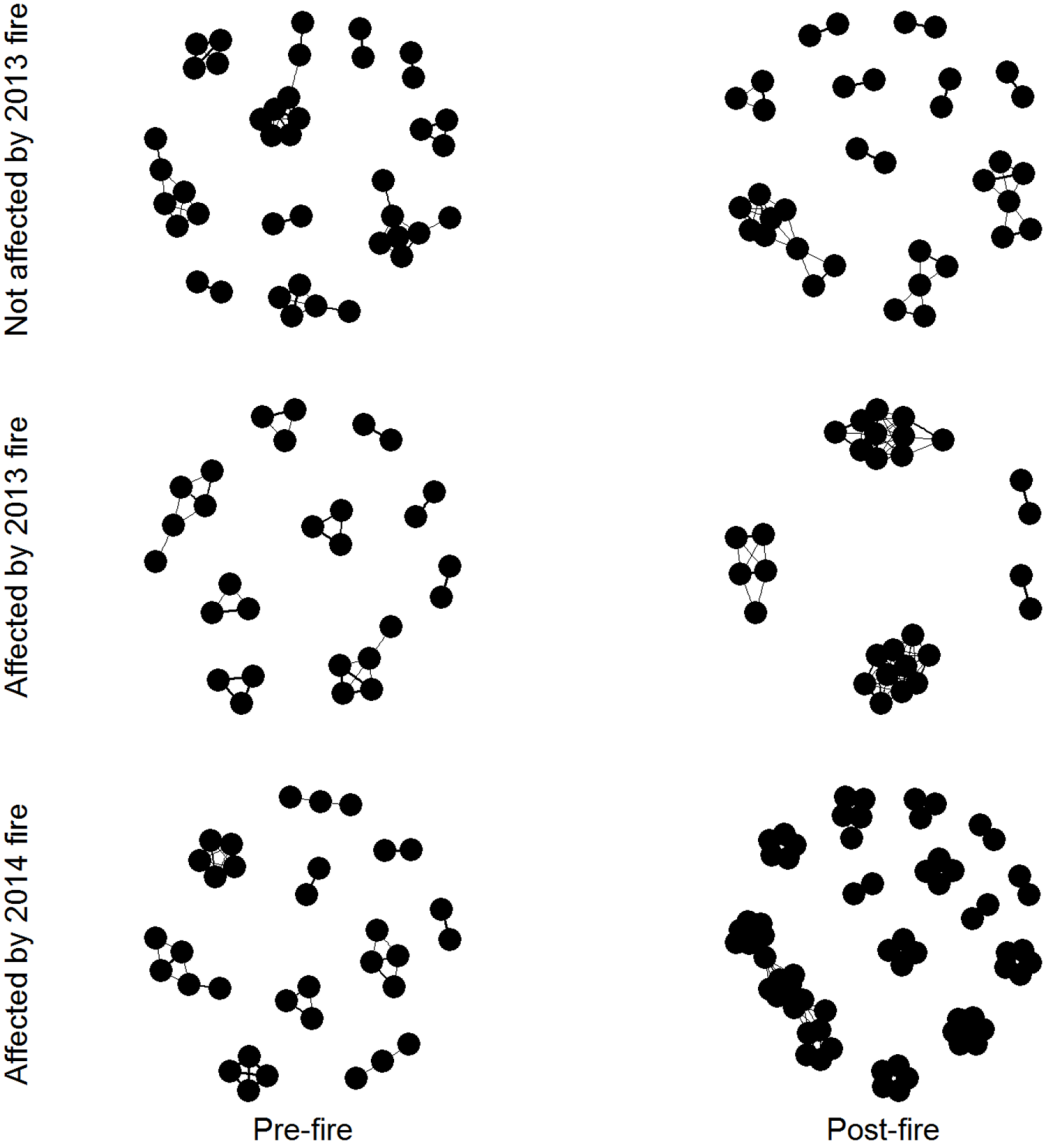
Social networks from 2013 and 2014 pre- and post-fire. Social networks in 2013 are separated by individuals that were affected by the fire and those that were not affected by the fires. Each individual bird is represented by a node, with social interactions represented by lines between them. Fire is associated with increases in social interactions with conspecifics in 2013 and 2014, as measured by weighted degree (seen in increased proportion of ties pre- to post-fire in 2013 and 2014 for affected birds), while birds that were not affected by fire in 2013 showed decreased connectivity after the fire.

**Fig 3:**
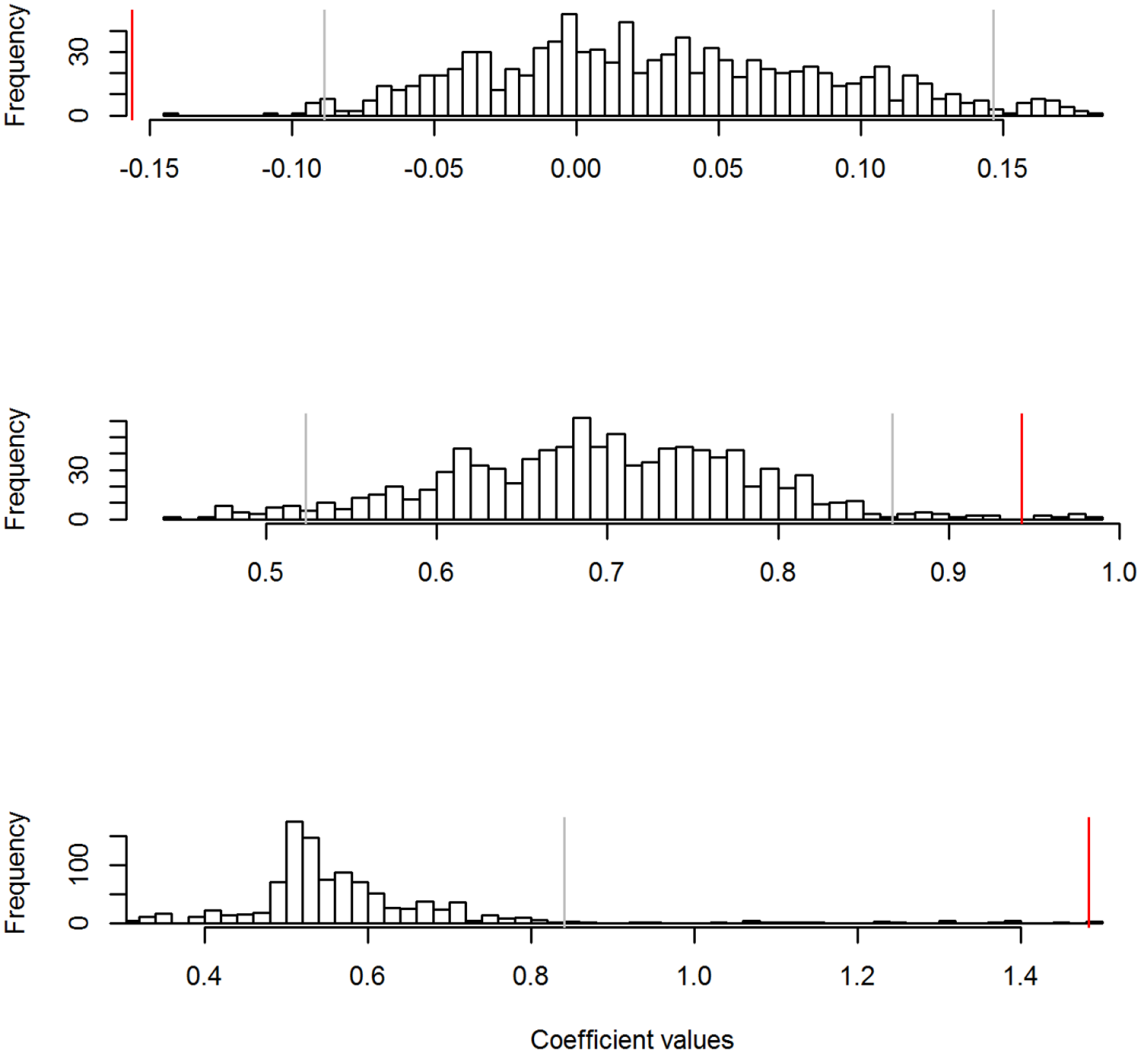
Observed model coefficients compared to a frequency distribution of coefficients from the same model based on randomized networks. Comparison of the coefficient value from the observed data (solid red line) to the frequency distribution of the coefficients from the same model based on randomized data via network permutations for a) 2013, fairywrens not affected by the fire, b) 2013, fairywrens affected by the fire, and c) 2014, fairywrens affected by the fire. In all three cases, the coefficient from the observed data is significantly different compared to the coefficients from randomized data. This coincides with higher weighed degree in 2013 and 2014 networks for birds that were affected by the fire, and lower weighted degree in 2013 for birds that were not affected by the fire.

We created four social networks in 2013 separating groups by temporal period (pre- vs. post-fire) and spatial location relative to the fire (affected vs. not affected), and two social networks in 2014 separating groups by temporal period (pre- vs. post-fire; Fig 2). Individuals affected by the fire had significantly higher weighed degree in the post-fire period relative to the pre-fire period in both years (2013: P = 0.007, 2014: P<0.001; Table 1, Fig 3). In contrast, in 2013 individuals that were not affected by the fire had significantly lower weighted degree in the post-fire period compared to the pre-fire period (P<0.001; Table 1, Fig 3). Higher degree in the affected network in 2013, in combination with lower degree in the concurrent unaffected network, suggests an effect of fire on social structure, rather than an artifact of temporal progression of the dry season. In 2013 the increase in group size was significant (P = 0.001), indicating that individuals using the observed data were in larger groups after the fire than expected. The change in group size was also significantly different than expected for individuals not affected by fire in 2014 (P = 0.001). However, in 2014, the increase in group size after the fire was not significantly different in the observed data than the permuted data, indicating that in this case higher weighted degree may have been due to tighter associations.

**Table 1:** Weighted degree of birds pre-and post-fire in 2013 and 2014.

	N	Weighed Degree (mean ± sd)
Affected 2013		
Pre-fire	28	1.41±0.57
Post-fire	29	2.35±0.96
Not affected 2013		
Pre-fire	41	1.44±0.58
Post-fire	35	1.28±0.32
Affected 2014		
Pre-fire	33	1.42±0.76
Post-fire	66	2.90±1.50

Observations in 2013 followed a Before-After-Control-Impact Design of birds both affected and not affected by fires, while all birds in 2014 were considered to be affected by fire. In 2013, individuals not affected by the fire had lower weighted degree post-fire compared to pre-fire. In both years of the study, individuals affected by the fire had higher weighted degree post-fire compared to pre-fire.

### Habitat change due to fire

**Fig 4:**
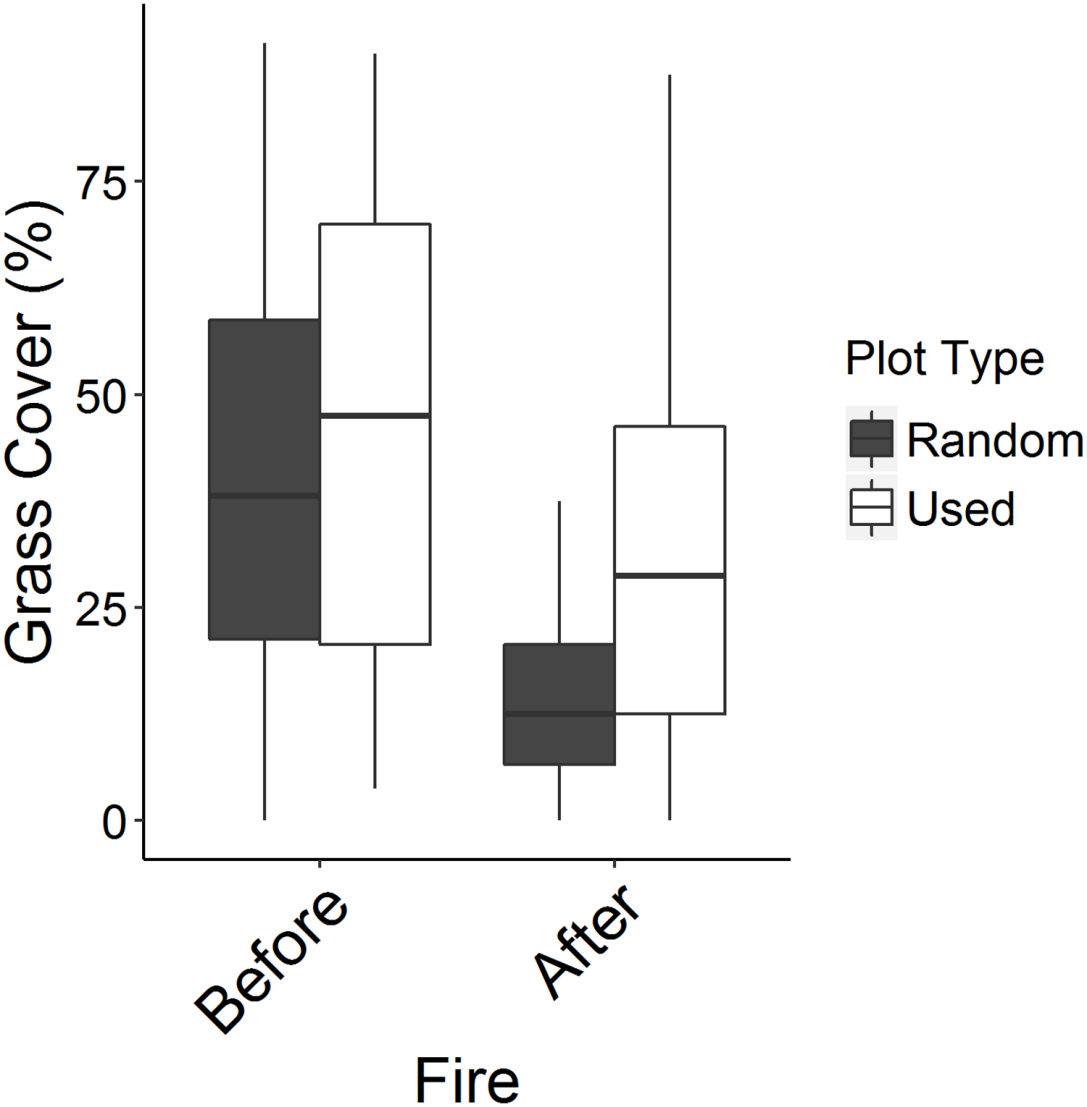
Pre- and post-fire grass cover in used and random plots. Red-backed fairywrens occupied areas with more grass (relative to random plots) following a bush fire in 2014. Before the fire, when the study site was characterized by higher grass cover overall, fairywrens chose sites that had similar grass cover to available sites. Fire reduced grass cover in both used and random sites after the fire relative to before.

In 2014, the only year that we conducted vegetation surveys, fire altered habitat structure by directly reducing the amount of grass habitat (W = 11311, P<0.0001, Fig 4). Before the fire, proportional use of grass cover by red-backed fairy wrens did not differ from random sites spread across the study site (W = 1129, P = 0.21), whereas after the fire, habitat used by fairywrens had more grass compared to random sites (W = 1359, P<0.0001, Fig 4).

## Discussion

This study adds a novel natural experiment to the growing body of literature on how the environment influences social structure by characterizing social networks that were directly impacted by fire before and after the fires and comparing them to 'control' social networks that were unaffected by fire. Among a resident population of red-backed fairywrens in northern Australia, we found that individuals had more social connections following fires than before fires, as determined by higher weighted degree. This change in social interactions was associated with birds in burned areas showing a preference for areas with disproportionately high amounts of grass following fires. In contrast, among individuals living in areas that were not affected by fire, and thus did not have any fire-associated habitat changes, we did not find a similar increase in social associations. This indicates that the increase in social interactions following fire was due to fire per se, rather than simply temporal change. These results support our prediction that, in this system, rapid environmental disturbance resulting in a change in resource availability serves to increase social interactions.

This study adds to previous research by demonstrating that in the case of large fires, shift in available habitat within the same relative area can result in a change in social interactions. Red-backed fairywrens selected habitat with more grass cover than control sites on the study area following fires. This finding is consistent with previous observations that individuals of this species whose home ranges were burned moved to neighboring patches of grass after the fires [28]. This, combined with increased social associations, indicates that red-backed fairywrens were congregating in vegetation that remained intact following fire events (see also [24], where lizards in areas with more frequent fires were geographically closer.) As such, increased flocking while remaining in the same area shows a flexible response to fire by red-backed fairywrens. A previous study also found that bush fires were associated with larger red-backed fairywrens groups [43], although this earlier study took place mainly post-fire in one year, did not measure habitat associations, and did not use a social network approach to investigate strength of social interactions.

There are several ways in which the increased sociality we observed in remaining habitat might have short-term ecological impacts on red-backed fairywrens. On our study site, insects are more abundant in areas with higher grass cover than burned areas [44], suggesting that red-backed fairywrens may be congregating in areas with more vegetation because of higher food availability relative to burned areas(see also [45,46]). Higher food abundance could either result in increased food availability or increased competition. Additionally, flocking can facilitate information transfer that can improve foraging efficiency [4], if fairywrens are moving between patches of grass based on insect abundance or availability. Another potential driver of increased flocking in remaining grass cover could be reduced risk of predation. Occupying a higher-density grass patch could reduce predation risk via increased access to suitable cover. Predators are often more numerous following fires, and some avian predators specialize on recently burnt areas [47]. Individuals with higher predation risk may benefit from increased social interactions [22] via a dilution effect [48] or increased predator detection [49] arising from higher relative densities of conspecifics, or some combination of these factors. However, there are also potential costs associated with increased sociality including increased risk of parasites or disease transmission (e.g., [2]), or increased competition for limited resources, which can lead to an increased risk of aggression [11,50]. At present, little information linking immediate changes in social structure to these down-stream effects, so identifying the short-term behavioral and ecological consequences of changes in sociality triggered disturbance therefore represents a promising area for additional research in this and other systems.

Our study utilizes behavioral observations of red-backed fairywrens in the context of large-scale fires as a natural experiment to characterize behavioral response to a reduction in available habitat. The finding that fire causes a reduction in grass is not surprising. However, increased strength of social interactions in areas that were affected by fire contrasted with decreased social interaction strength in areas that were not affected demonstrates a key linkage between habitat disturbance and behavior. Generalizing beyond fairywrens, when non-lethal disturbance has the effect of reducing available habitat or resources (e.g., fire; grazing [51], deforestation [52]) and/or creating more clumped patches, we would expect that density of individuals using remaining patches may increase in the short term (e.g., [53]). This in turn should lead to increased social interactions within remaining patches or at high concentrations of remaining resources, as was the case with this study.

We must also acknowledge several potential caveats associated with this study. First, our study site has a red-backed fairywren population density that is substantially lower than that at other sites where this species has been studied (e.g., [54,55]), thus our sample size is relatively small and our social networks are constructed from limited numbers of observations, which can make estimating social associations difficult [56]. Nonetheless, we consider our findings, particularly those associated with the Before-After Control-Impact aspect of the study, to be robust. Despite having relatively small sample sizes, the relative uncertainty should be the same between treatments (pre- and post-fire, and control vs. impact), and weighted degree is more robust to testing than other network metrics such as graph density. Although comparing networks of different sizes can be problematic [57], we believe that the increase in weighted degree in individuals is biologically meaningful (see [13]) and not simply an artifact of increased sample size in the post-burn treatment, which is supported by the lack of an increase in weighted degree in control birds despite similar changes in sample size. Additionally, we collected data in the same ways across treatments, and the permutation tests that we used should control for sample size. Improved technology allowing for more continuous tracking of social interactions, as well as similar studies conducted at sites hosting higher densities of this species, would allow us to determine how broadly applicable our results are. For example, it is possible that the increase in social interactions we report would not occur in habitat that is already saturated; conversely, it is possible that results may be even more exaggerated in a higher density population. Also, because of limited breeding at our field site during the two years of study, we have limited data on reproductive success of individuals in the population, and thus are unable to assess how the changes in social structure we documented might influence individual fitness. Investigating these possible carry-over effects represents another priority for future research in this system.

A more thorough understanding of individual social behaviors and responses to environmental stressors can be critical for conservation and management of social species [58], particularly because of high levels of anthropogenic environmental disturbances (e.g., forest fragmentation [59]), coupled with climate change, which can alter habitat along a number of different axes [60]. Our study is one of the first to examine social response to an environmental stressor by looking at the same population before and after an environmental disturbance that altered available habitat. This study joins a number of studies across taxa in finding flexible social structure that can respond to changes in habitat complexity or other ecological factors [12,14,26]. Future work should focus on how this alteration of social structure can influence fitness, for example by influencing sexual signals or mate choice decisions.

## Supporting information

S1 Table Red-backed fairy-wren group associations. Observations of red-backed fairywren groups of color-banded birds in 2013 and 2014 are provided in a.csv table. Spatial period denotes whether groups were observed pre- or post-fire, and location was used to determine if individuals were affected by fires.
(CSV)
